# Working together to co-produce better health: The experience of the Collaboration for Leadership in Applied Health Research and Care for Northwest London

**DOI:** 10.1177/1355819620928368

**Published:** 2020-06-02

**Authors:** Cicely A Marston, Rachel Matthews, Alicia Renedo, Julie E Reed

**Affiliations:** 1 Professor of Public Health, London School of Hygiene and Tropical Medicine, UK; 2 Programme Manager, National Voices; 3 Assistant Professor, London School of Hygiene and Tropical Medicine, UK; 4 Strategic Director, CLAHRC NWL, Patient and Public Engagement and Involvement Lead, National Institute for Health Research (NIHR) Collaboration for Leadership in Applied Health Research and Care for Northwest London, Imperial College London, Chelsea and Westminster Hospital, UK; 5 Visiting Professor in Improvement Science, School of Health and Welfare, Halmstad University, Sweden

**Keywords:** patient involvement, participation, dialogue

## Abstract

**Objectives:**

To improve the provision of health care, academics can be asked to collaborate with clinicians, and clinicians with patients. Generating good evidence on health care practice depends on these collaborations working well. Yet such relationships are not the norm. We examine how social science research and health care improvement practice were linked through a programme designed to broker collaborations between clinicians, academics, and patients to improve health care – the UK National Institute for Health Research Collaboration for Leadership in Applied Health Research and Care for Northwest London. We discuss the successes and challenges of the collaboration and make suggestions on how to develop synergistic relationships that facilitate co-production of social science knowledge and its translation into practice.

**Methods:**

A qualitative approach was used, including ethnographic elements and critical, reflexive dialogue between members of the two collaborating teams.

**Results:**

Key challenges and remedies were connected with the risks associated with new ways of working. These risks included differing ideas between collaborators about the purpose, value, and expectations of research, and institutional opposition. Dialogue between collaborators did not mean absence of tensions or clashes. Risk-taking was unpopular – institutions, funders, and partners did not always support it, despite simultaneously demanding ‘innovation’ in producing research that influenced practice.

**Conclusions:**

Our path was made smoother because we had funding to support the creation of a ‘potential space’ to experiment with different ways of working. Other factors that can enhance collaboration include a shared commitment to dialogical practice, a recognition of the legitimacy of different partners’ knowledge, a long timeframe to identify and resolve problems, the maintenance of an enabling environment for collaboration, a willingness to work iteratively and reflexively, and a shared end goal.

## Introduction

To improve health care provision, there are times when academics are asked to collaborate with clinicians, and clinicians with patients. It is widely believed that obtaining good evidence on practice depends on these collaborations working well.^1,2^ Yet such collaborative relationships are not the norm, and how exactly they should be established is far from clear.^3^ In this paper, we examine how social science research and health care improvement practice were linked together within a Collaboration for Leadership in Applied Health Research and Care (CLAHRC). CLAHRCs were consortia funded by the United Kingdom’s (UK’s) National Institute of Health Research to broker collaborations between clinicians, academics, and patients to improve health care, and to help close the ‘second translational gap’, i.e. to have everyday health care practice informed by research.^2^ Each CLAHRC in England developed its own approach on how to ensure research informed health care practice.^4^

The existing research shows that the co-production of knowledge within partnerships for health care improvement is difficult to achieve. Some CLAHRCs operated in a ‘command and control’, top-down fashion and others had more distributed power and decision making.^2^ Three CLAHRCs emphasized knowledge generation over implementation and research, and implementation teams tended to be siloed^2^. In some CLAHRCs the knowledge co-production arising from the interaction between the different types of knowledge brought to the table by the partners was limited.^5^

Understanding how collaborative relationships between partners might achieve high-quality knowledge co-production is crucial.^5^ ‘Boundary spanning’ is vital: experts enhance their activities by forging relationships external to their normal sphere of operation, making connections outside the usual boundaries of their professional community.^2,6^ This is fundamental to cross-disciplinary work and creating the dialogue between different types of knowledge brought by partners to co-produce knowledge and implementation strategies sensitive to local health care contexts.^2,6^ It is also important to clarify what co-production in health care means in practice, and what it is that is being co-produced through collaborative relationships.^7^

The challenges of implementing research findings within complex health care settings are well known, although solutions are not usually forthcoming^1^. Nevertheless, co-production of knowledge by researchers and research users has been identified as a key ingredient for successful health care improvement.^1,8,9^ With some notable exceptions, such as a study of a researcher-in-residence model,^5^ little is known about the micro-processes governing whether or not co-production occurs, and how co-production can be supported.

In this paper, we investigate how co-production of knowledge can be encouraged and sustained within collaborative partnerships, complementing and adding further detail to the existing work on CLAHRCs. We draw on social psychological theory of partnerships to discuss successes and challenges of collaboration. We also consider our own experiences of partnership working in the nexus between social science research and health care improvement teams to advance knowledge and practice in patient and public engagement and involvement (PPEI) within the CLAHRC for Northwest London. We suggest ways to develop synergistic, rather than oppositional, relationships between partners that encourage and enhance knowledge co-production.

## Social psychological theory of partnerships

Aveling and Jovchelovitch define ‘partnership’ as ‘a situated encounter between the different knowledge systems of concrete partners’^10 (p.35)^ involving processes of interaction and communication between familiar (own) and unfamiliar (partners’) modes of thinking, which are dependent on the sociocultural and institutional characteristics of the partnership context.

As ‘complex, evolving systems of social interactions’^10 (p.35)^ and ‘ongoing practice’,^10 (p.41)^ partnerships require ‘explicit focus, critical reflection and an enabling institutional context’.^10 (p.42)^ People come to partnerships with different knowledge, identities, resources, histories, experience, and understandings. The way a partnership evolves is shaped by how partners perceive each other, their knowledge, their willingness and ability to take others’ perspectives, and engage with others’ knowledge.^10^

Understanding partnerships requires attention to: (1) partnerships as knowledge encounters, (2) representations of the self and other, (3) styles of communication, and (4) representational projects (i.e. how each party represents their group’s interests).^10^ ‘Transformative’ partnerships based on dialogical practices, critical reflection, and mutual and reciprocal relationships of empathy involve partners engaging with what they see as legitimate differences, as opposed to the ‘monological’ approach of imposing their own perspectives. In partnerships there is space for debate, and the tensions between different types of knowledge and opinions can lead to enhanced knowledge outcomes^10^. Trust,^11^ a recognition of interdependencies between partners’ contributions,^12^ and resources to support the collaboration are also important.^11^

## The collaboration

The collaboration we examine here was between social science researchers and health care quality improvement (QI) research-practitioners (staff who were tasked with facilitating QI while conducting research into the use and impact of QI approaches). It occurred within the CLAHRC for Northwest London (CLAHRC-NWL), one of the^13^ CLAHRC funded by the UK’s National Institute for Health Research. CLARHC-NWL used a pragmatic approach, integrating QI methodology (e.g. action-effect diagrams and plan-do-study-act-cycles) with PPEI.^13^ The social science research team from the London School of Hygiene & Tropical Medicine (LSHTM – CM, AR) partnered with the CLAHRC’s health care implementation and QI team (JR, RM) to provide academic insights from in-depth qualitative research into the PPEI work of CLAHRC-NWL. Chelsea and Westminster Hospital NHS Foundation Trust and Imperial College London were awarded a grant to fund the consortium of over 20 NHS, academic, and third-sector partners. The grant paid for a leadership team located in Chelsea & Westminster Hospital/Imperial College (the CLAHRC-NWL team), which subcontracted partner organizations, including the LSHTM.

During the first phase (2008–2013), CLAHRC-NWL funded, trained, and supported QI projects carried out by teams of multidisciplinary NHS frontline staff. Teams had to learn about and use QI methods, and operate in line with CLAHRC-NWL’s overall ethos. This included involving patients and carers in QI work, translating research into better care, undertaking multidisciplinary collaborative work, and collecting data as a matter of routine for rigorous evaluation.^14^

We have previously discussed how the organizational culture of CLAHRC-NWL helped to engage patients and carers meaningfully in QI projects,^14^ and the underpinning philosophy of the programme.^8,9,13^ We have also produced evidence on the nuances and complexities of PPEI.^14^^–16^ This paper examines the role of the collaboration between LSHTM (represented by CM and AR referred to here as the ‘social science team’) and CLAHRC-NWL (represented by RM and JR, referred to here as the ‘implementation and quality improvement (IQI) team’) in co-producing this PPEI knowledge and advancing practice.

## Methods

All data included in this study were contributed by the authors. We generated qualitative data about the collaboration using a reflexive dialogue exercise between members of the two collaborating teams, in which we explored the topic of our collaborative relationship. We used open questions as prompts to facilitate critical dialogue between the social science and IQI teams. The dialogue started via a 3 h one-to-one meeting (January 2017) between the two groups (led by JR), followed by written and verbal exchanges further exploring and refining understanding on issues raised at the meeting. The participants are all named as authors here. Similar dialogical reflexive approaches have been used previously to examine collaborative practice in knowledge co-production.^17^ During the dialogue, we attempted to clarify each other’s perspectives and produce useful insights to help us understand our collaboration and help others, by uncovering the facilitators and pitfalls that we encountered and bringing the intangible to the surface. The dialogue reflected on our 10 years of collaboration, which included four years of ethnographic work.

Our analysis was informed by Aveling and Jovchelovitch’s^10^ framework and examined the interaction between diverse identities, forms of knowledge, and resources brought by each of us to the collaborative encounter, identifying key themes and developing them into the findings presented here. Quotes presented below are from the discussion and iterative writing, with some edits for clarity.

We also used an additional data source to help us analyse the collaboration. This was AR/CM’s four years of ethnographic work which involved interacting with the IQI team in order to research the PPEI within the CLAHRC-NWL. The ethnographic research involved observations and interviews. It used interpretivist grounded theory, was iterative, and was a theory-building approach, aiming to add depth to understanding of PPEI and community participation in health.^14,18,19^ As part of this ethnographic ‘slow research’,^20^ AR/CM also aimed to understand our position as participant observers of CLAHRC (including the IQI team) and reflected on how our relationship with CLAHRC (and JR and RM/the IQI team) changed over time and shaped the data collection we were carrying out during our investigations into PPEI^21,22^ and its interpretation/manifestation within CLAHRC-NWL.

Our study was approved by London NHS REC (National Research Ethics Committee) 09/H0718/35.

## Findings

## Challenges to knowledge systems and identities

The study revealed key elements explaining why collaborations might succeed or fail in working together and sustaining relationships. The theme of risk was central to how experiences of collaboration for knowledge co-production were framed, and particularly the role of institutions and spaces in supporting the risk-taking and experimentation necessary for successful collaborative knowledge co-production.

At the start, the collaboration was assisted by a mutual desire to improve participation in health care and research (entailing dialogue, mutual respect, and progress). In Aveling and Jovchelovitch’s terminology, we shared a ‘representational project’. The IQI team wanted to change practices within health care settings to make them more responsive by using research in practice and strengthening PPEI. The social science team wanted to find better ways to understand community and PPEI participatory interventions (by developing new research methodologies and new theoretical understandings of participation in health, including PPEI). However, the partnership immediately challenged our knowledge systems and identities. NHS jargon, for instance, was often incomprehensible to the social science team, and the interpretivist ethnographic approach of the social science team contrasted with the positivist knowledge systems (e.g. emphasis on evaluation and impact) within health care.

The QI work was designed to be responsive and iterative, with ongoing experimentation to see what would work and how. The different timescales required for practical changes in the programme versus academic theory building of the social science team’s grounded theory approach soon became apparent. The IQI team wanted rapid and pragmatic feedback on PPEI to improve their work – something the social science team did not feel able to provide:

We didn’t know what ‘success’ of the PPEI work was supposed to look like, from the CLAHRC point of view, which made formal evaluation difficult. I think sometimes now we forget how little in-depth work had been done on participation at the beginning. (Social science team member)

An important part of transformational dialogue is partners engaging with what they see as legitimate differences in ways of thinking and working rather than imposing their own perspective.^10^ For instance, early on the social science team argued that while simple analyses of patient engagement could be done over a few months, a longer timeframe was essential for more in-depth work. To the social science team, the IQI team’s willingness to accommodate this seemed respectful and recognized the academic expertise they brought. Meanwhile, the social science team respected the IQI team’s ability to transform the health care environment and inspire others to do so.

The presence of dialogue between the partners did not mean the absence of tensions or clashes. Learning how to handle these carefully was part of the process. For instance, one IQI team member spoke of initial feelings of falling short in some areas where they had limited experience such as understanding academic writing. Some of the solutions, including ‘becoming more robust’ also applied to working with patients:

I’ve become more tolerant of that discomfort … Sometimes if you have a bit of a thinner skin you’re quite sensitive to what others are thinking and feeling, and that’s a real component of some of the work around PPEI: the sensitivity you need to really understand where people are coming from. But then the other side of it is that’s actually not always that helpful. Sometimes you need to be a little bit more robust. (IQI team member)

## Unequal risk-taking

At first, the two teams worked in parallel. The social science team conducted in-depth sociological work to develop theory and produce high-quality outputs for academic journals, while the IQI team developed and conveyed a vision of what the overall CLAHRC-NWL programme should be. The IQI team also conducted PPIE activities in an environment often hostile to such approaches.

In this period the social science team unknowingly capitalized on the risks taken by the IQI team. At the outset, it was unclear whether the CLAHRC activities, bridging research and practice, would be accepted as rigorous enough (by academia) or rapid enough (by practitioners). This tension reflected the competing expectations of university and public sector organizations. By occupying the middle ground between these traditional institutions, members of the IQI team were risking their career progression because their own research outputs might not meet the traditional institutional demands of academia. The IQI team expended time and energy building relationships and spaces for discussion and learning, to create what we now see as protected spaces. The CLAHRC leadership team conducted political negotiations under the auspices of the NIHR funding award to create these spaces for thinking and experimentation which were somewhat protected from traditional institutional demands. This allowed others to work according to the CLAHRC-NWL vision and benefitted the social science team both by creating a rich environment that was interesting to study, and by leaving space for the social science team to pursue academic leads without requiring specific types of outputs.

The discomfort – but potentially high reward – of the CLAHRC-NWL approach of iterative improvement departed from the status quo and was met with institutional resistance, for example with IQI team members under pressure for not publishing fast enough or in the ‘correct’ journals. Senior academics within the IQI team’s institution framed their expectations of outputs and success criteria in ways that did not always take account of the purpose of the NIHR CLAHRC funding (to translate evidence into practice to improve patient care), or the appropriate approaches for this type of work (such as the need for participatory approaches engaging frontline staff and patients). Members of the IQI team felt at risk in their individual careers because the activities they considered vital were not accounted for within traditional academic criteria for promotion and advancement – a risk compounded by their employment on fixed term rather than indefinite contracts.

At the time, the social science team were only dimly aware of these difficulties, while nevertheless being grateful for the space that the CLAHRC-NWL team allowed for in-depth exploration and ‘slow’ research over the years of the programme.^23^ This ‘slow’ approach was needed to capture temporalities and unexpected aspects of QI in health care, such as how the nature of patient involvement in QI projects changed over time, how patient input influenced the programme, and vice versa, and how this mutual influence and impact evolved over time.^16,24^

## Creating a shared representational project

The IQI team used a strategy of continual learning and improvement for health care, explicitly valuing learning how to have better dialogues, including with patients and carers.^14,25^ This approach also helped develop and maintain collaborative relationships with a range of people and organizations:

PPEI just resonates with the whole programme…PPEI is not something that’s separate…The principles that underlie it are key to how we do the whole programme, about enabling people to contribute and collaborating. And I see the challenges of engaging patients are just as much as between engaging a nurse and a doctor. (IQI team member)

To achieve progress, the collaboration, but particularly the IQI team, had to go through a ‘process of sense-making’, involving persuasion and negotiation, as well as constantly rearticulating and applying the ‘CLAHRC vision’ to establish shared ways of doing things (dialogue, reflexivity, improvement) with partners outside the IQI team. This was to ensure that these principles were applied to all aspects of the programme, not just PPEI:

How do you work with individuals who all have individual drivers and motivations and capabilities and inspire them towards a bigger vision that you’re all working towards? It’s kind of like: we want to be over here because we think that’s the right place to be or best place to be, but you’re here. So can we encourage you to go a little bit here? Or to take a step in this direction? Or…Oh, no, you think going over there’s better? All right, yes, well, no, maybe that is better. There’s that constant…co-creation. But at the same time having a clear vision. And it feels, to be honest, quite exhausting to maintain it. (IQI team member)

As the collaboration matured, so did the shared identity and shared understanding. The framing of the social science team as externally ‘evaluating’ PPEI also reduced and eventually disappeared. A new shared understanding emerged of the value of integrating rapid testing cycles to help practitioners working at pace and in complex settings, while also generating theory to guide sustainable and meaningful change. This was possible because of the duality inherent in the collaboration. For instance, the IQI team was interested in evaluating the merit of specific strategies (such as a patient representative training programme), while the social science team was interested in understanding the programme in depth (such as investigating identity processes, spatial aspects of PPEI). As one of the social science team commented: ‘The freedom and trust we were given created an enabling environment for in-depth exploration and critical theory development’.

Insights from qualitative research conducted by the social science team^7,14^–^16,18,19,23^ gradually became part of the dialogue of the CLAHRC-NWL collaboration. Over time, the IQI team also started to use more of a qualitative and ethnographic approach.^13,25^ Meanwhile, the core purpose of improving health care through more meaningful participation helped anchor the work:

I felt that holding the vision and thinking about where we are going was my main job [in the first five years], whereas now I feel that is really distributed amongst the team and we’re much more co-producing our future vision. (IQI team member)

## Creating a ‘potential space’ where innovation and change can happen

We borrow the phrase ‘potential space’ from Winnicott’s^26^ work on children’s development and Jovchelovitch’s^27^ work on knowledge making where the potential space, or space-in-between, is a space where one feels safe to explore and be creative. Winnicott uses it to refer to a transitional space between mother and child, where the child can start experiencing the world through play and be creative while being safe.

We repurposed it here to refer to a somewhat analogous process where participants move from monological, non-experimental practice and the insular world of their original disciplinary communities, to practice that is participatory, outward-looking, dialogical, experimental, and reflexive.

A key strength of the collaboration was the creation of a potential space^26,27^ between each partner’s original, nascent identity, knowledge, and projects. This and the fact that the CLAHRC NWL was a new, evolving entity, allowed experimentation with new forms of acting and thinking, including engaging with others’ knowledge so that members of the collaboration could think freely about how to improve services or engage patients, or develop theory. Maintaining the space took a great deal of effort and entering it could require a change in one’s worldview or practices:

[More effective ways to improve the NHS and do research] take time, they change power hierarchies, they change relationships, they require time and investment, so how do you get people to move from that really reactive, knee-jerk, target-driven culture to something much more collaborative and productive? (IQI team member)

The IQI team saw their space as transformative for the people within it. Both the IQI and social science teams use dialogical approaches to teaching and mentoring, rather than didactic approaches, using a reflexive, critical pedagogy not commonly seen in medical schools. The IQI team frequently spoke of having a core desire for transformative action: that CLAHRC-NWL should promote critical awareness, and support personal change to help transform workplaces and health care. This required a safe space in which the origins (knowledge and identity) of the partners were recognized and understood as legitimate and where mutual transformation was possible. If one side simply told the other to change, while resisting transforming themselves, this would imply a monological approach.

For the IQI team it was exhausting to try to strike this balance between accepting people as they are, while also staying true to the deep commitments to the programme. Having supportive relationships at the heart of the programme helped with the emotional strain of maintaining this central space.

### Creating a potential space within academic institutions

The collaboration had to overcome a number of institutional barriers to create the potential space. Both teams felt that the extensive mentoring needed to develop new ways of working and to facilitate open dialogue required substantial effort, yet this type of labour was not specifically rewarded or acknowledged by the teams’ host institutions. For instance, the social science team had to ignore their academic institutions’ usual measures of success (such as producing many articles in high-impact medical journals), even if this was regarded by the institutions as unsatisfactory performance: ‘The only option really is constant “failure”, but not caring. Why do you keep going? Because the status quo is unsatisfactory’ (Social science team member).

Celebrating successes helped create a supportive environment that encouraged further experimentation. One aspect of this was what an IQI team member referred to as making the intangible (the work they were doing to create space for dialogue and participation) tangible (i.e. within a peer-reviewed publication documenting successes). While both teams were under pressure to publish in ‘high impact’ medical journals, the social science team wished to explore the collaboration in a way that was more suited to social science journals. The IQI team had not expected this approach, and the social science team had not anticipated that the IQI team would value it, yet the space created by CLAHRC NWL allowed these less predictable outputs to emerge that were valued in unexpected ways:

There’s like an academic weight behind the [PPEI] theme [of CLAHRC-NWL]. There’s something that gives it some validity and credibility, and I know when the [social science team] papers have been published it’s made me feel much stronger and much more confident in talking around the [PPEI] theme. And then there’s that reciprocal relationship between the fact that your paper’s drawn on the work that we’ve done and then we draw back on the work that you’ve done. (IQI team member)

When institutional demands clashed with demands on the collaboration, we sometimes failed to come to a satisfactory resolution. The individualistic focus of academia (e.g. only one person can be ‘first author’ on a paper, even if contributions by all the authors are crucial) hindered true collaborative working because however much we believed in the overall project and wanted to spend more time on collaborative actions, our jobs were insecure if we stopped producing specific types and quantities of journal articles. This meant that we had limited time to develop the more experimental collaborative work and felt pressure to contribute traditional outputs (primarily first-authored, peer-reviewed journal articles).

Our desire to collaborate and trust that others were acting in good faith helped us to keep working together and to try to resolve clashes. Over time, our collaborative working was helped by using dialogue to understand the constraints and challenges we each faced in our institutions, which helped give context to contributions and shortcomings.

## Discussion

In our experience working on the CLAHRC-NWL, the different interests and systems of knowledge produced mutually valuable and reinforcing outputs, creating synergies that advanced both teams’ understanding of PPEI for health care improvement. That is to say, there was co-production of knowledge. The skills within the collaboration – which developed, iterated and improved as the PPEI work matured – were crucial for strengthening the entire collaboration. For example, both teams worked together with patients who were involved in CLAHRC-NWL to co-create the research project ‘This Sickle Cell Life’, which further developed better integration of the views and experiences of patients and members of the public, which is important in improving health care quality across the board.^23,28^

### Five key elements for success

Five core elements helped maintain the collaboration, all related to developing and sustaining meaningful dialogue:

**Shared identity and understanding:** These two crucial ingredients for collective mobilization in the context of participation^29^ helped us to navigate through clashes and misunderstandings. This created a strong potential space to experiment with different ways of working and to engage in productive dialogues, theoretical advances, and health care QI.

**Respect for each other’s expertise and willingness to listen**: Even when we had institutional demands that seemed to contradict what would be good for the collaboration, our dialogues helped us to keep the collaboration going. The type of reflexivity facilitated through these dialogues is a useful way to facilitate collaboration for knowledge co-production.^21^ Aveling and Jovchelovitch^10^ cite case studies where clashes between different accountability structures were overcome via Dialogue Seminars which helped each partner understand others’ perspectives, commitment, etc. During our reflective discussion, we repeatedly returned to the importance of dialogical approaches, including mentoring and facilitating, as well as the crucial point about trusting partners to act in good faith.

**Positive reinforcement:** Academic ‘feedback’ from the social science team provided positive feedback for the IQI team on the process of PPEI, where otherwise the focus was primarily on health care activities that could have gone better. This, in turn, provided additional perceived legitimacy to the project. From the social science team side, the positive reinforcement was instrumental, in that the team received money and freedom from the primary grant-holder (CLAHRC NWL) to continue the work, and ontological, in that their raison d’etre and work were recognized through action taken by the IQI and health care teams.

**Willingness to take risks:** Working within a space where risk-taking is normalized can be motivating in itself, in that it signals the possibility of changing the status quo. Academia and health service providers value traditional markers of success. Individuals’ willingness to take risks, to trust and to compromise, had to overcome institutional or cultural pressures to maintain the status quo. Individuals’ willingness to take risks also helped strengthen the relationship between the teams.

**Normalization of dialogue and experimentation**: Oppressive rules and institutional structures were avoided as much as possible. There were sometimes issues with disciplinary hierarchies (or perceived hierarchies) or other status markers that needed to be addressed to allow dialogue to happen and trust/collaboration to progress. Many of the problems in our collaboration stemmed from intense institutional pressures that seemed designed to stop us from working in new ways. Similar challenges to knowledge co-production emerging from competing institutional demands have been found elsewhere.^21^

Of course, institutional expectations must be carefully managed because without institutional support, collaboration will likely fail.

## Limitations

There are two main limitations to our study. First, we do not claim our experiences are universal. Each collaboration will face its own unique set of challenges and opportunities. Nevertheless, we hope the general points we raise here can be adapted and used to assist in other collaborations.

Second, we are reporting on our own collaboration. This means that, while we have been as reflexive as possible in our observations, we may be unaware of key components of the collaboration. The advantage of our reflexive account is that it is based on nearly 10 years of working together and has the advantage of insider knowledge that would be hard to obtain otherwise.

## Conclusions

Within health care there is a sense that ‘getting research into practice’ requires novel solutions and synergies that can be found when different people with different expertise collaborate.^11,30^

We suggest that successful collaboration involves creating a potential space where experimentation and synergies can emerge. Yet, as we have shown here, spaces to achieve genuine collaboration require both monetary and more nebulous forms of support, which are often not available. Risk-taking is unpopular: institutions, funders, and partners do not always support it, despite often simultaneously demanding innovation. Part of having a genuine potential space for experimentation is having time to think and the freedom to try to do things differently. We need to be serious about collaborative working if we are to solve perennial problems. This means ensuring there are resources to create and maintain potential spaces, which will allow the risk-taking required for creativity and knowledge co-production. This, in turn, will lead to innovative, targeted, and sustainable solutions to emerge.
